# Healthy family traditions and personal health assets – salutogenic resources for oral health among young adults in vulnerable communities in South Africa: a qualitative study

**DOI:** 10.1186/s12903-025-06941-z

**Published:** 2025-09-23

**Authors:** Marie Nordström, Ulrika Lindmark, Eva Wolf, Tshakane Ralephenya, Deliwe Mtyongwe, Angelique Kearney, Pumla Sodo, Yolanda Malele-Kolisa

**Affiliations:** 1 Faculty of Odontology, Malmö University, Malmö, Sweden; 2 Department of Health Sciences, Karlstad University, Karlstad, Sweden; 3 Department of Community Dentistry, University of the Witwatersrand, Johannesburg, South Africa; 4 Faculty of Health and Environmental Sciences, Central University of Technology, Bloemfontein, South Africa; 5 Department of Family Medicine and Primary Care, University of the Witwatersrand, Johannesburg, South Africa

**Keywords:** Salutogenesis, Health promotion, Socioeconomic status, Oral health

## Abstract

**Background:**

Dental caries is a global public health problem with persistent inequalities. Research with a salutogenic perspective, as in, a focus on health factors, can provide important knowledge to be used in health promotion. The aim was to explore salutogenic resources among dental caries-free young adults living in vulnerable communities in South Africa.

**Methods:**

A total of 32 participants (28 females, 4 males, mean age 26.2 years) with no previous caries experience were purposively recruited from two under-resourced townships. The qualitative data from interviews were audio-recorded, transcribed, and analyzed via qualitative content analysis.

**Results:**

The resulting theme, *A salutogenic foundation for oral health: preservation of traditions and use of personal health assets as protection against challenges*, comprised two categories: (1) Individual health assets and early intergenerational learning, and (2) Ability to apply learned health strategies. Having individual health assets and tools for coping, early learning experiences by positive family influence, being exposed to healthy traditions during hardships, and the ability to apply learned health strategies were important salutogenic resources. Together, these resources formed a salutogenic foundation for oral health which enabled individuals to develop healthy routines, make healthy choices for oral health, and maintain oral health when encountering challenges and hardships.

**Conclusions:**

Salutogenic resources for oral health empowered individuals from vulnerable communities to maintain oral health. This suggests that future health promotion interventions should be considered and directed at multiple levels, targeting individual, family, community, and structural factors to promote sustainable oral health.

**Supplementary Information:**

The online version contains supplementary material available at 10.1186/s12903-025-06941-z.

## Background

Dental caries is a global public health problem that not only has a large impact on quality of life, by causing discomfort, dental pain and infections, but also contributes to high economic costs for both individuals and society [1]. In South Africa, over 90% of young adults in a low socioeconomic population are affected by dental caries [2]. The prevalence of dental caries is influenced by social determinants of health, following a social gradient with worse oral health among low socioeconomic groups [3]. The worst affected third of the South African population has nearly three times higher caries rates compared to the average [4]. South Africa is recently reported to be one of the most unequal countries in the world with 55% of the population living in poverty [5]. The unemployment rate is high among young individuals, at 45.5%, and living conditions are tough, with 13.6% living in informal settlements. In addition, nutrition related challenges include nearly 20% of the population experiencing inadequate access to food [6] and high obesity rates, with 31% of men and 64% of women diagnosed as overweight or obese (BMI ≥ 25 kg/m^2^) [7].

The salutogenic theory involves a focus on health factors rather than risk factors, and it is suggested for oral health promotion [8]. While poverty and health inequalities remain challenges in South Africa, some individuals are free from dental caries, despite their low socioeconomic status (SES). Applying a salutogenic perspective is a way to study how some people maintain health despite the challenges they may face in life. The availability of health-supporting factors in individuals and their environment, combined with an ability to utilize them, serves as salutogenic resources that strengthen health [9].

The importance of salutogenic resources for oral health has previously been highlighted [10–12], but few studies have been conducted in low SES populations or among young adults. Among children from a low SES background, salutogenic resources such as sense of coherence (SOC) [13], family resilience [14], and social support [15] were important. Previous studies that analyzed qualitative data of young adults showed that parental support and guidance, internal resources, and supportive environments were important for maintaining and promoting oral health [16–19]. The development of individual salutogenic resources is influenced by the availability of supportive environments such as social and cultural support [20]. This may explain why countries with different cultures and levels of socioeconomic development may nevertheless experience good oral health. This calls for studies that take place in contexts other than those previously explored. To our knowledge, no earlier studies exist that have explored salutogenic resources contributing to the development and maintaining of oral health among individuals of low SES in South Africa.

Research using qualitative data with a salutogenic perspective is a way forward to gain a deeper understanding of health-supporting factors. The insights gained can be applied to developing both future oral health promotion and health promotion, with an emphasis on empowerment and sustainability to reduce health inequalities [8]. The aim of this study was therefore to explore salutogenic resources among dental caries-free young adults living in vulnerable communities in South Africa.

## Material and method

This study follows a design suitable for analysis of qualitative data, using an inductive approach, and is reported according to COREQ (Consolidated criteria for reporting qualitative research) [21]. The study was approved by Human Research Ethics Committee (Medical) in South Africa, reference number M201106. Written informed consent, in English, was obtained from all participants.

### Context of the study

Decayed, Missing or Filled Teeth (DMFT) among young adults in a low socioeconomic population in South Africa was 6.8 among 18–24 year-olds, 10.3 among 25–34 year-olds, and 15.8 among 35–44 year-olds (2014–2016) [2]. South Africa has a dentist-to-population ratio of 1:8900 [22], in comparison to a high-income country such as Sweden, where the ratio is approximately 1:1300 [23]. In addition, the public sector in South Africa provides service for 80% of the population, but only employs 20% of the dental workforce [22].

The recruitment took place in two low socioeconomic communities in South Africa: Diepsloot in Johannesburg, Gauteng Province; and Rocklands in Mangaung, Free State Province. These vulnerable and under-resourced areas are often characterized by overcrowding; poor access to electricity, water, and sanitation facilities [24]; and often a higher caries prevalence [4].

Diepsloot is an informal settlement on the outskirts of Johannesburg. The population is young, with an average age of 25 years. The unemployment rate is 30.2%, and only 48% have access to the main electricity supply [25]. Although data on young adults is not available, dental caries prevalence among children and adolescents in Gauteng was 25.9% in the permanent dentition and 30.2% in the primary dentition in 2017/2018 [26].

Rocklands is a township dwelling situated in Mangaung municipality. The municipality is the most unequal in the Free State, with an unemployment rate of 25.3% and 36.6% living in poverty. In 2016, 18.9% of households in Mangaung have not had enough money to buy food during the last 12 months [27]. While there is no data on young adults, the most recent data shows that caries prevalence levels among 15-year-olds in Free State was 54.5% (1999–2002) [28].

### Participants

Participants were purposively recruited at two community clinics situated in Diepsloot and Rocklands. Inclusion criteria were young adults ≥ 18 years and no teeth affected by dental caries (DMFT = 0). The upper age limit for young adults in South Africa is 34 years [6], but can be considered to be up to 45 years according to an adult development perspective [29]. Participants were identified in person by approaching individuals attending the community clinics for non-dental related issues. A dental screening calibration exercise was carried out on five persons, followed by DMFT assessment by five of the authors (AK, DM, PS, TR, YMK) according to criteria of the World Health Organization [30]. A total of 37 individuals were approached, 22 in Diepsloot and 15 in Rocklands. Five individuals were excluded due to not meeting the inclusion criteria or declining participation. Three individuals with DMFT scores of 1, 2, and 5 respectively were interviewed. As their DMFT score were lower than the previously reported mean for the age group in the studied population, it was decided to include the three individuals. Finally, 32 participants were included —28 females and 4 males—of mean age 26.2 years (range 18–43 years). All except three individuals were free from dental caries experience. The majority had, at most, a high school education (68.8%), and more than half (53.1%) were unemployed (Table 1).

**Table 1:** Background characteristics of participants

	Diepsloot, Johannesburg (*N* = 17) *n* (%)	Rocklands, Mangaung (*N* = 15) *n* (%)	Total (*N* = 32) *n* (%)
Age			
18–25 years	12 (70.6)	8 (53.3)	20 (62.5)
26–35 years	2 (11.8)	6 (40)	8 (25)
36–43 years	3 (17.6)	1 (6.7)	4 (12.5)
Sex			
Women	15 (88.2)	13 (86.7)	28 (87.5)
Men	2 (11.8)	2 (13.3)	4 (12.5)
Civil status*			
Single	11 (84.6%)	13 (86.7%)	24 (85.7%)
Married	2 (15.4%	2 (13.3%)	4 (14.3%)
Dental caries status			
DMFT = 0	14 (82.4)	15 (100)	29 (90.6)
DMFT ≥ 1	3 (17.6)	0 (0)	3 (9.4)
Considers own mouth being healthy*			
Yes	10 (76.9)	15 (100)	25 (89.3)
No	3 (23.1)	0	3 (10.7)
Occupation			
Care worker	3 (17.6)	2 (13.3)	5 (15.6)
Student	4 (23.5)	1 (6.7)	5 (15.6)
Unemployed	5 (29.4)	12 (80)	17 (53.1)
Other	5 (29.4)	0 (0)	5 (15.6)
Highest education level			
College/University	2 (11.8)	8 (53.3)	10 (31.3)
High school	14 (82.4)	6 (40)	20 (62.5)
Primary	1 (5.9)	1 (6.7)	2 (6.3)
Self-rated socioeconomic status*			
High	0	0	0
Medium	5 (35.7)	5 (35.7)	10 (35.7)
Low	9 (64.3)	9 (64.3)	18 (64.3)

*Missing values

### Data collection

After receiving oral and written information about the study and written informed consent, participants also filled out a short questionnaire regarding participants considering their own mouth being healthy (Yes/No), occupation, highest educational level (No formal schooling/Primary school/High school/College/University), and self-rated socioeconomic status (Low/Medium/High) (Table 1). Each of the five authors (AK, DM, PS, TR, YMK) first conducted a pilot interview during October and November 2021, followed by feedback on the interview technique, including relevant follow-up questions, by the authors MN and UL. The pilot interviews were not included in the analysis. The final interviews were conducted at the community clinics during March 2022 in the language of each participant’s preference and performed by the author proficient in the same language. Participants were recruited until no new relevant information for the research question could be obtained from new participants. The languages employed by the study included English, Sesotho, Sepedi, Zulu, Xhosa, and Shona. The interviews were semi-structured and explored salutogenic resources in everyday life, both current and in childhood. The interview guide was inspired by a similar study in Sweden [18], which was translated, piloted, and then adapted. The opening question was “Tell me about your day yesterday, what did you do? Try to tell as much detail as possible”. Other questions touched upon unusual days, special days, grocery shopping, eating habits, childhood, and family. Follow-up questions were asked to allow the participant to develop, clarify, and reflect. The full interview guide is available (Appendix 1).

The interviews were audio-recorded, transcribed *verbatim*, and translated into English if applicable by an external researcher. The translations were verified by back translation and, if needed, corrected by five authors (AK, DM, PS, TR, YMK). Each interview lasted between 6 and 24 min, with a mean length of 16 min. Only the participant and the researcher were present during the interviews.

### Research team and reflexivity

The research team had a background in odontology and oral health (four dentists, three dental hygienists, and one dental therapist), including varied research experience (two without previous research experience, one master’s student, three PhD students, one PhD, and one professor). Four of the authors (MN, UL, EW, PS) had previous experience of conducting studies using qualitative data. Based on previous studies [18, 31], the preconception was that the origin of salutogenic resources among low SES populations shows a complex picture that also varies in different contexts.

### Data analysis

The analysis was carried out using qualitative content analysis (QCA) according to Graneheim and Lundman [32]. It followed an inductive approach to broadly explore salutogenic resources, where the authors performed multiple readings of the transcripts to gain a sense of the whole. Meaning units were identified followed by condensing (shortening the text while keeping the context intact). One author (MN) then coded and sorted the codes according to their similarities and differences. By abstraction, the codes were categorized to describe the visible (manifest) content. The identified pattern was discussed, and the categories and subcategories were modified. A theme representing the red thread and the underlying meaning (latent content) was developed, followed by discussion and modification by all authors. Quotes, translated into English, were used to illustrate the theme, thus identifying the pattern.

## Results

**Fig. 1:**
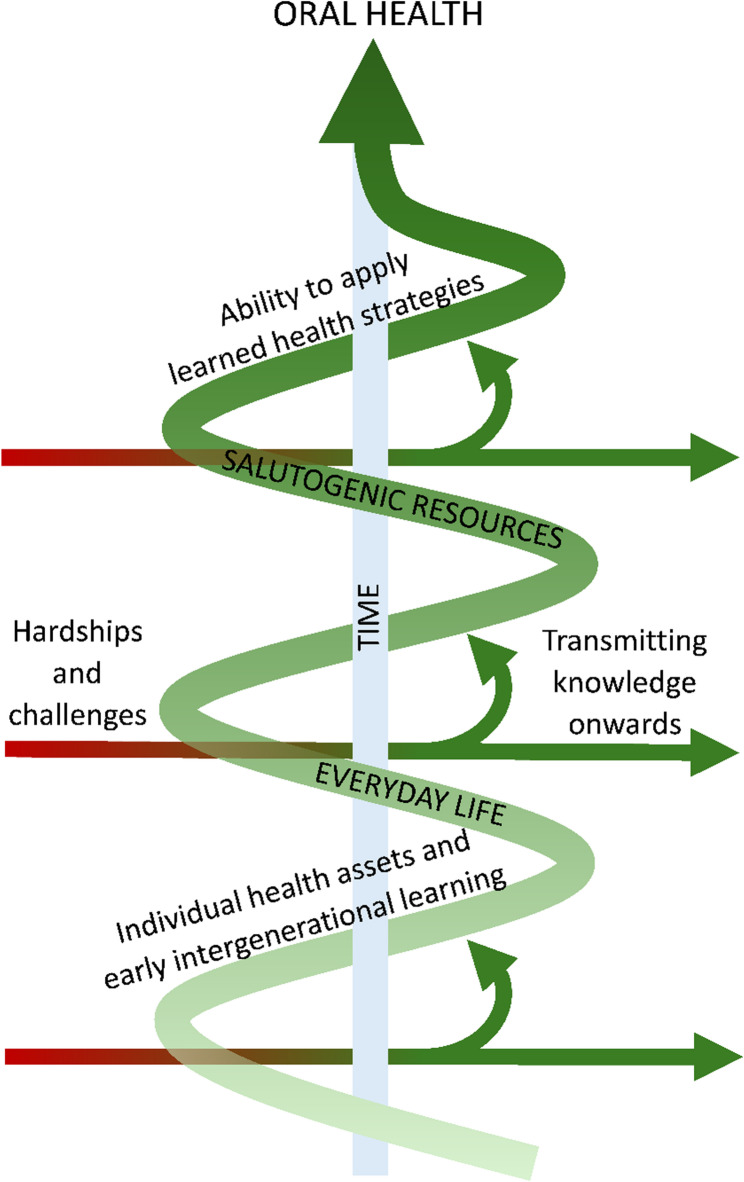
Illustration of the results. The analysis showed regularity in everyday life while there was a constant change in the food environment over time and during the life span (central arrow). The encounter of hardships and challenges enters everyday life (red arrows). By protection from salutogenic resources, they return as strengthening experiences for health that are also transmitted onwards to others (green arrows)

The analysis revealed a theme, *A salutogenic foundation for oral health – preservation of traditions and use of personal health assets as protection against challenges*. The theme encapsulates the importance of one’s health assets, experiences of early intergenerational learning, and ability to apply learned health strategies (Table 2). These salutogenic resources created pathways to maintaining oral health when participants encountered hardships and challenges. In addition, simultaneous changes in the food environment during the participants’ lifetime were described (Fig. 1).

**Table 2:** Theme, two categories, five subcategories, and examples of codes

Theme	Category	Subcategory	Example codes
A salutogenic foundation for oral health –preservation of traditions and use of personal health assets as protection against challenges	1. Individual health assets and early intergenerational learning	I. Tools for coping and acceptance	Finding ways within the limitations imposed by poverty Appreciation during difficult times Social support for tough times
		II. Exposure to healthy traditions during hardships	Accustomed to traditional food during upbringing Economic hardships force traditional, healthy food
	2. Ability to apply learned health strategies	III. Balancing restrictions and routines	Stable food routines with variations Exceptions for special occasions Structured plans for grocery shopping
		IV. Being motivated for healthy choices	Self-regulating behavior; Conscious food choice with health in mind Inner driving force to healthy habits
		V. Transmitting knowledge onwards	Long-term thinking for children’s health Striving to be role model for good oral health Tooth brushing as part of daily routines for oneself and children

### Category 1: individual health assets and early intergenerational learning

Participants described first-hand experiences of hardships, such as poverty, COVID-19, unemployment, abuse, or losing a parent. Having individual health assets and social support from family and people nearby or in the neighborhood enabled participants to face the challenges – *Tools for coping and acceptance* (Subcategory I). Being exposed to traditional food, with a low content of sugar and processed food, as children and maintaining it into adulthood was another health resource – *Exposure to traditions during hardships* (Subcategory II).

#### Subcategory I: Tools for coping and acceptance

One limitation imposed by poverty is that food is not always readily available. During struggles, the participants described eating only simple meals consisting of *pap* (maize porridge) and milk or *morogo* (African spinach), meals in smaller portions, or even no food and thus hunger when going to bed. However, certain strategies were used to adapt, including being creative with what was bought and prioritizing important basic supplies that last longer and can be sufficient to make a simple meal, such as oil and mielie meal (maize). Participants also described excluding or reducing meat or unhealthy food items, such as sweets, snacks, and fast food, or looking out for special offers and buying cheap, locally grown vegetables.

“We don’t eat too much when we are (uhm) on a difficult patch. […] We eat pap, spinach and chicken feet. We eat spinach because my mum has a garden close by. But when there is money, we eat meat.” (Participant 23).

During their upbringing, the participants’ parents made an effort to hide their struggles by creating a good atmosphere at home or occasionally providing food out of the ordinary. Having company, sharing joyful moments, and receiving consolidation from family or friends are described as important ways of coping. Sharing experiences about life and their different concerns, as well as cooking and eating together, was a way to tackle and forget the hardships.

The energy to cope could also come from finding internal support from religion or beliefs and going to church, which was an important source of strength and confidence when there were challenges in life.

“I would say that I usually did not see [hard times] because we were a lot of adults, and they make sure that we don’t see that there is something wrong, covering. We grew up as if we not struggling […] I force things so that my children don’t see that [we] don’t have money, just the way I grew up as well, I hide it.” (Participant 28).

Positive coping took the form of meeting challenges and struggles by having acceptance of the situation or what has passed, having tolerance, or thinking that better times will come. Some of the participants described showing acceptance of, and even gratitude and appreciation for, the simple food that was available and trying to make the best of it.

“Now she [mother] works only for two days, so yeah, we have to maintain the budget. […] We have to cut some things and buy basics, the things that we use, [or] need the most. I have accepted that, because it is temporary, I know.” (Participant 17).

During economic difficulties, social support could be important. The participants described growing up with experiences of support from family, relatives, neighbors, or friends who could help and provide basic food supplies. Another example was when mothers in the neighborhood would lend money to help each other out. Participants described how suddenly no income came in during COVID-19 or after losing a job. During these circumstances, social security was an important safety net for providing basic food supplies. In addition, school was a valuable place for the provision of hot meals.

“There were difficult times when we were young when we had to go ask for mealie-meal and African spinach from other people. Even pap was difficult to get. There was no meat. Even soup was difficult.” (Participant 6).

However, negative coping when dealing with stress or boredom or being weighed down by thoughts was also described. In those situations, access to food could be a way to regulate and handle the negative feelings.

#### Subcategory II: exposure to healthy traditions during hardships

Participants described experiencing positive food influences during upbringing such as being served home-cooked “traditional food” by parents or grandparents. The traditional food included pap and milk, beans, morogo, *mahewu* (fermented, liquid mealie-meal porridge), *Mopane* worms, cabbage, and peanuts. Parents and grandparents gave positive attitudes towards healthy food by conveying health messages and instructing participants about what types of food to eat. These exposures impacted participants to be accustomed to, enjoy, and appreciate the traditional food. They maintained the same dietary habits from childhood into adulthood, even expressing feeling awkward when not eating traditional food. The participants gave positive descriptions of their traditional food culture and how traditional food was an integral part of daily life, including having home-grown vegetables or seeing their animals running freely in the garden or being able to drink fresh cow’s milk. The preservation of traditional food thus also had the positive outcome of eating healthily.

“Growing up and being in my family, they have been cooking a certain type of food. I have never been exposed to other types of food. Now I am not able to. I can’t even try other food, because I am used to what I grew up eating. I can’t even change it.” (Participant 7).

Eating traditional healthy food was also a way to make ends meet when encountering economic hardships. Access to a garden or land provided the possibility to farm, grow their own vegetables, and keep their own animals for meat or milk, without being dependent on money or having the pressure to earn money. In contrast, the participants’ adult life in the cities could be harsh, as there was no access to land for farming. Food was only available by buying it, with no way to acquire it when out of jobs or unemployed.

“When we were struggling, we were just eating pap, maize, peanuts, beans, watermelons. All traditional foods, because we farmed those foods at the villages. We make mealie meal with maize [and] cook pap or porridge with it.” (Participant 25).

In times of poverty, participants experienced how their parents still enforced oral hygiene but when toothbrush or toothpaste was not available, ashes and water were used instead. Parents increasingly bought local vegetables or staple food to cook homemade food that lasts longer. Drying vegetables could also be a way to preserve them for future use and save money. Hence, not having access to money reinforced the maintenance of simple traditional homemade dishes. Unhealthy food, including processed food, sweets, snacks, and fast food, was eaten to a limited extent due to its unaffordability and bought mostly on special occasions, when making exceptions, or when feeling cravings. The traditional food was thus not necessarily a conscious choice. However, when money was available, it became a matter of personal choice and an opportunity to splurge on fast food or unhealthy food.

“It’s food that we afford, and it lasts. Money makes us choose. For sure, we’d be eating fast foods every day, but because the money is not there, we will buy mealie-meal, knowing that it will push us a month, a bit.” (Participant 31).

Some participants described changes in the general food environment where junk food and pre-packaged processed food were described as being more readily available nowadays compared to the time of participants’ upbringing. Processed food, sweets, snacks, and fast food such as *kota* (sandwich filled with both meat and french fries), fat cakes (fried dough), and chips were types of food more recently encountered. They wanted to eat the “new food”, especially after not being able to buy it during financial struggles. Therefore, fast food is seen as a luxury or desirable. At the same time, participants were aware of the unhealthiness of modern food and its adverse effect on dental health. They could reflect on the healthiness of the traditional food that they were exposed to during childhood by parents and grandparents. These were experiences that helped them to make healthy choices and have healthy habits in adult life.

“The time when I was living with my sister was difficult. Even if you crave fat cakes, it was difficult to buy. But now, when I crave a fat cake, I can go buy myself. […] whatever you crave, [you] eat it because you have been struggling.” (Participant 14).

### Category 2: ability to apply learned health strategies

Positive learning experiences and influences during upbringing and early life gave the participants the ability to apply learned health strategies. They had habits to create a balance in everyday life – *Balancing restrictions and routines* (Subcategory III). Participants had a sense of purpose and capability for their daily lives – *Being motivated for healthy choices* (Subcategory IV). Finally, participants not only used their knowledge but also passed it down to their own children as well as others – *Transmitting knowledge onwards* (Subcategory V).

#### Subcategory III: Balancing restrictions and routines

Participants described how most days looked the same with regularity and consistent routines. Food in everyday life did not have large variation. It was often pap or rice, vegetables, and meat. Positive learning experiences and influences on routines from parents and grandparents, such as getting accustomed to eating healthy traditional food during upbringing, persist into adulthood of the participants.

“We eat pap almost every time. If not pap, it will be rice, or spaghetti, macaroni. The only that will differ will be what kind of relish, *seshebo*, we eat. Red meat or sometimes chicken, or mincemeat. We do eat vegetables maybe once or twice in a week.” (Participant 19).

During unusual days, weekends, special occasions, and celebrations, the participants reported eating not necessarily unhealthy food but other types of food, dishes, or types of meat not eaten normally, although sometimes cold drinks, cake, snacks, and sweets were also consumed. This signifies a balance between the healthier food during usual days and unhealthy food on other occasions.

“An unusual day, it’s when I am under pressure with work. I have a lot of assignments to do. I don’t have much time for myself. After work, get home, eat, sleep. On a normal day, I do eat proper food, healthy food like rice, vegetables and fruits, I don’t like pap, or spaghetti. But on an unusual day, I end up eating not proper food, biscuits, Simba (crisps), sweets.” (Participant 27).

The participants’ routines for grocery shopping were the result of observing and following in the footsteps of parents or relatives. The shopping was structured and planned by checking what is needed beforehand and making and following a list. Although what the participants could buy was restricted by a tight budget, the money could last longer with a plan, and unhealthy food was also bought less. Exceptions were made for sweets or junk food, which was bought outside the list to satisfy cravings.

“I go according to the list, according to my budget. […] First, I’ll start with mealie meal, for pap, for mealie-meal for papa. Rice, then the meat section. After, the vegetables just to balance. After, if I have a little bit more money left, then I will come out of the budget a bit. That usually happens often. I get tempted at times.” (Participant 15).

Routines for oral hygiene could start during childhood. Oral hygiene aids in adulthood included toothbrush and toothpaste with the toothbrush being changed on a regular basis as it is worn out. Toothbrushing was described as part of daily hygiene routines such as bathing or connected to waking up, going to bed, or having a meal.

“I make sure that I brush my teeth before bathing. I bath in the morning and in the evening, meaning I brush my teeth in the morning and evening.” (Participant 24).

#### Subcategory IV: being motivated for healthy choices

Participants expressed motivation, meaningfulness, and different ways of resourcefulness when it came to their health behavior or attitude toward health. They had an inner driving force to promote their general health by making conscious choices or striving toward routines. When realizing they had bad general health, for example, being overweight, having high blood pressure, or experiencing other negative effects on their quality of life, they could make active choices and efforts to promote their health. When the new healthy routines were established, resulting in noticeable improvements in health, they described this as rewarding and took pride in their efforts.

“Three years back, I came to a point where I need to eat healthier than before. […] Then I started eating differently […] I lost weight [and became] much healthier. [Now] I am able to run, able to do certain things that I was not able to do.” (Participant 20).

Participants reflected with awareness and insight on their dietary habits and nutritional intake, and how these can affect the body. In addition, the participants also described being able to self-regulate by consciously restricting their intake of unhealthy drinks or foods to avoid bad health.

“Even if I crave nice things, I tell myself that what I am going to buy, I am going to buy… Things that is not there, in my mind, I don’t do that.” (Participant 26).

Participants described how they could gain oral health-related knowledge from family members or healthcare and use the knowledge in practice. However, participants expressed lack of knowledge and insight into the causes of oral diseases, but with a desire to learn more. The motivation to practice good oral hygiene and tooth brushing could be for socializing or having a nice breath and appearance.

“The teeth is the most connecting thing. Someone sees you, then she sees the teeth first. […] I always make sure that I brush three [times per day].” (Participant 21).

#### Subcategory V: transmitting knowledge onwards

The participants described how they passed on routines, knowledge, economical thinking, and positive influences to their own children or parents. By including the children in their own toothbrushing and dietary habits, healthy routines were passed on.

“At the park, I buy him [son] fruits like apples. I buy him Simbas (crisps), I don’t buy him many sweets because I fear that his teeth might get rotten.” (Participant 12).

The passing on of this information could also go beyond their family and children. Participants showed that they wanted to be good role models and had ambitions to promote the oral and general health of others by conveying health messages and encouraging others to practice good oral hygiene.

“We came for an interview about teeth. I will encourage people out there that, let us brush our teeth twice a day, in the morning and evening. Then your teeth will remain healthy, clean.” (Participant 2).

Participants saw the consequences of unhealthy food, both for oral and general health. They realized that unhealthy food had negative effects on their children’s health. With their children’s or parents’ long-term health in mind, they not only provided healthy food and meals but also limited the amount of unhealthy food or attempt to create boundaries.

“I think the traditional foods are healthy. You have to enjoy eating traditional food, don’t eat the luxurious food, like sweets, chocolates, they might damage your teeth. […] I am not going to give her [daughter] chocolates. She likes to eat too much. I am going to help her.” (Participant 22).

## Discussion

This study explored salutogenic resources for oral health in a vulnerable community in South Africa, and the following salutogenic resources were found: having individual health assets and tools for coping, early learning experiences by positive family influence, being exposed to healthy traditions during hardships, and an ability to apply learned health strategies. Together, these resources formed a salutogenic foundation for oral health which enabled individuals to develop healthy routines, be motivated, make healthy choices, and maintain oral health when encountering challenges in life.

The participants in this study can be seen as capable and resource strong. They experienced significant challenges and hardships, such as poverty, but were able to adapt and cope during those difficult times, indicating resilience and the ability to adapt in positive ways in the face of adversity [33]. In addition to coping, feelings of appreciation and acceptance were expressed, suggesting optimism and expectations of positive outcomes in the future. Studies have shown the importance of both coping and optimism for good oral health among young adults [34]. Using resources and being able to successfully adapt to hardships can be an empowering experience which also enhances SOC [9, 35], a concept sometimes used in health promotion, as mentioned further below.

Among the participants, social support by family and friends was an important safety net and important for coping and creating security in times of economic distress. Social capital, as in, social resources from social structures and individual networks creating trust and support, has been highlighted as important for oral health [36]. Support from the local community or neighborhood can act as an important stress buffer and create a sense of belonging and safety [37]. Systematic reviews have shown that both community and individual social capital have a protective effect on oral health outcomes in children and adolescents [36] as well as among adults [38]. High neighborhood empowerment was associated with lower dental caries rates among adolescents [39, 40]. Future interventions suggest including community based approaches with the potential to strengthen social capital and trust, and thereby the empowerment and oral health of the people in the community [41].

Another important aspect for handling poverty in this sample was farmland and social grants for secure access to food. In South Africa, 11.6% of the population experiences hunger [6]. In addition, around a quarter of the population receive social grants [24], which can be the main source of income for some [42]. Participants were sometimes able to address the food insecurity in difficult times by growing their own vegetables. Households practicing agriculture are more likely to be with food secure as well as have a more diverse diet [24]. However, today in South Africa, only 13.8% of households are involved in agriculture [6]. Taken together, this highlights the importance of access to farmland and social grants, which may increase the possibility of healthy food choices, with limited amount of sugar and processed food, suggesting more research is needed within the area of health promotion action.

The participants in this study preserved food traditions and indicated that they are proud of their culture. Although many of these traditional foods have a low status and are associated with those who have low socioeconomic status [43], the participants had been exposed to traditional food during their upbringing, and despite its low status, maintained a positive view of the traditional food, also described as healthier than modern food. This is in line with an earlier study of parents of Indigenous children in Ecuador which found that limiting processed food and maintaining traditional food, such as food harvested from the family farm, was a protective factor against caries among the children [44]. Less frequent consumption of processed sugar- and starch-containing foods is associated with lower dental caries experience [45]. Additionally, adolescents from industrialized nations like Sweden have stated that the accessibility of unhealthy food and sweets impacts their decisions regarding nutritious versus non-nutritious food choices [17]. Preserving culture by serving traditional food could be a key to health but also aid in achieving food security [42]. Although unhealthy food, including processed food, sweets, snacks, and fast food, was often readily available, participants often had limited financial means to buy unhealthy food. Previously, parents from low-income townships in South Africa described experiencing challenges in the accessibility of unhealthy food around the home [46]. This strengthens the argument that policies and regulations should address the commercial determinants of availability and pricing, for example, by decreasing the cost of healthy food and increasing the cost of unhealthy foods, thus facilitating healthy choices [47].

In this study, shaping healthy behaviors and routines was facilitated by being exposed to family support and routines. In this regard, dental care was not mentioned by the participants. The results from a study among healthy young adults from a low socioeconomic background in Sweden show similarities regarding both the importance of parental support and guidance in fostering individual salutogenic resources and the limited role of dental care [18]. Previous studies both in Sweden [17] and South Africa [48] show that parents play an important role in creating a positive home environment, being good role models, and forming and establishing healthy dietary behaviors. Therefore, introducing habits and routines during childhood by targeting the health promotion efforts of parents may increase the chance of establishing lifelong healthy behaviors.

The participants in the current study had an inner driving force, motivation, engagement and an ability to self-regulate and reflect. These resources can be related to the SOC components of meaningfulness, manageability, and comprehensibility [9], indicating a strong SOC among participants. SOC, and its components and resources for healthy choices influencing oral health, has also been discussed based on results from interviews with Swedish 19-year-olds [17]. Persons with a strong SOC can identify and use resources to a greater extent [9]. Higher SOC has also been shown to be associated with healthier oral health habits and oral health status, which is also the case in the South African context [49]. Salutogenic interventions including strengthening of resources have been suggested [50]. Previous interventions show promising results regarding the strengthening of SOC, even in low SES populations [51, 52], which in turn could potentially improve social capital and coping [38]. In addition, a person-centered, theory-based approach, in which the patient has an active part in the treatment, such as when using motivational interviewing as one communication tool, has the potential to improve oral health-related behaviors [53]. Therefore, opportunities are open in different arenas to strengthen individual salutogenic resources using psychological theories, to promote and maintain good oral health. However, more knowledge and evidence from long-term follow-ups are needed, especially in relation to dental caries as outcome [54].

The participants expressed that they are both the recipients and transmitters of knowledge and routines. They take responsibility for their own health as well as act as a kind of health ambassador. An empowered individual can be a health ambassador with the potential to spread knowledge to their family and those in the neighborhood. Interventions among parents and caregivers have the potential to spread not only to their children but also to others in the community. Conveying knowledge to others also enhances self-belief and individual empowerment [55]. Previous studies show that neighborhood empowerment had a protective effect on caries experience, potentially by the spread of information by members in the local community and the enforcing of positive behavioral norms [39, 40]. Instead of top-down approaches, participatory strategies in which members of the local community are involved in designing and performing interventions, with the potential to emphasize the local culture, could be the way forward [56].

### Strengths and limitations

A study design utilizing qualitative data enables a deeper understanding of health factors related to different local contexts. To find dental caries-free individuals, sampling was carried out at community clinics, which might limit transferability to a general population. A majority of the participants were female, which means that gender-specific results might have been overlooked, for example which salutogenic resources that had more relevance for the individual [35] and depending on what sex the individual identifies as. Although it was desirable to recruit young adults with a balance between different gender, it was not possible to continue recruiting participants as the study took place during COVID-19 and its associated restrictions. Another possible weakness of the study design included interviews conducted in different African languages, which required translation and thus the risk for misinterpretation. However, the authors conducting interviews were proficient in the languages and could conduct the interviews in the language preferred by the participant and also verify the translations.

The study also contains strengths, and measures were taken to ensure trustworthiness. First, the data was collected from two sites, which increases confirmability [57]. In addition, pilot interviews were conducted to improve interview technique as well as to test the interview guide in the current context. Data was collected until it was deemed that no new information was gained from interviews, thus, saturation was assessed to have been reached [58]. All authors were involved in the analysis, and their expertise has been provided, increasing dependability [59]. Information on the background characteristics of the participants, as well as the study context, has been provided to assess transferability. Examples of codes and quotes were provided to ensure credibility [59]. The figure illustrating the results can be related to the salutogenic theory and life course perspective, thus showing the results in a theoretical context.

Persistent oral health inequalities necessitate new approaches instead of traditional preventive measures that only focus on risk factors. In line with the World Health Organization recommendations [60], the results from this study in South Africa suggest that health promotion interventions need to be considered and directed at several levels: strengthening salutogenic resources on the individual level by, for example, person-centered approaches; working at the family level by targeting parents and caretakers; and on the community level by using participatory approaches and addressing structural factors by policies and regulations. This can be related to the influences on health, as described in the model by Dahlgren and Whitehead [61], and it is also in line with the Ottawa Charter for Health Promotion that recommends developing personal skills, strengthening community action, and creating supportive environments [62].

## Conclusions

Salutogenic resources for oral health were participants’ individual health assets and tools for coping, early childhood experiences shaped by positive family influence, exposure to healthy traditions during hardships, and the ability to apply learned health strategies. These constituted a salutogenic foundation for oral health that empowered individuals from vulnerable communities to establish healthy routines, make healthy choices, and maintain oral health when encountering challenges and hardships.

## Supplementary Information

Supplementary Material 1.

## Data Availability

The data generated and analysed during the current study are in the repository at the Department of Community Dentistry, School of Oral Health Sciences, University of the Witwatersrand, Johannesburg, South Africa and is available on reasonable request to Tshakane Ralephenya: tshakane.ralephenya@wits.ac.za.

## Abbreviations

COREQ: Consolidated criteria for reporting qualitative research
DMFT: Decayed, Missing or Filled Teeth
QCA: Qualitative content analysis
SES: Socioeconomic status
SOC: Sense of coherence
