# Supplementary material for: Healthy family traditions and personal health assets – salutogenic resources for oral health among young adults in vulnerable communities in South Africa: a qualitative study
Source: BMC Oral Health. 2025 Sep 23;25:1418. doi: 10.1186/s12903-025-06941-z (PMC12459066; doi:10.1186/s12903-025-06941-z)
Supplement: Supplementary file 1 — Supplementary Material 1. [file 12903_2025_6941_MOESM1_ESM.docx]

**Appendix 1. Interview guide**

**Opening question**: Tell me about your day yesterday, what did you do? Try to tell in as much detail as possible.

| **Additional questions** |
| --- |
| Tell me about your typical day?  How would an unusual day be/look like? |
| On a normal day, what do you usually eat? |
| When you have special occasions, what do you normally eat? |
| When you go grocery shopping, how do you decide what to buy? |
| When you were growing up, what is that you would normally eat? |
| When you/your family were going through a difficult time, what would you normally eat? |
| How did your family influence your current eating habits? |
| Daily, what made you decide what to eat? |

**Finish**: Is there anything else you want to tell me? Is there anything else you are thinking about?

**Follow-up questions will be asked for clarification, reflection, and in-depth answers, for example:**

Can you tell me more?

Can you give an example?

What were your thoughts then?

How did it affect you?

How come you did that?

How did you feel then? How did you think then?
